# Development and validation of risk prediction model for post-donation renal function in living kidney donors

**DOI:** 10.1038/s41598-024-61107-1

**Published:** 2024-07-05

**Authors:** Seong Jun Lim, Jieun Kwon, Youngmin Ko, Hye Eun Kwon, Jae Jun Lee, Jin-Myung Kim, Joo Hee Jung, Hyunwook Kwon, Young Hoon Kim, Jae Berm Park, Kyo Won Lee, Sung Shin

**Affiliations:** 1 Division of Kidney and Pancreas Transplantation, Department of Surgery, Asan Medical Center, University of Ulsan College of Medicine, 88, Olympic-ro 43-gil, Songpa-gu, Seoul, 05505 Republic of Korea; 2 Department of Surgery, Soonchunhyang University Seoul Hospital, Soonchunhyang University College of Medicine, Seoul, Korea; 3 Division of Transplantation, Department of Surgery, Samsung Medical Center, Sungkyunkwan University School of Medicine, 81 Irwon-Ro, Gangnam-Gu, Seoul, 06351 Republic of Korea

**Keywords:** Kidney, Glomerulus

## Abstract

This study aimed to create and validate a predictive model for renal function following live kidney donation, using pre-donation factors. Accurately predicting remaining renal function post live kidney donation is currently insufficient, necessitating an effective assessment tool. A multicenter retrospective study of 2318 live kidney donors from two independent centers (May 2007–December 2019) was conducted. The primary endpoint was the reduction in eGFR to below 60 mL/min/m^2^ 6 months post-donation. The primary endpoint was achieved in 14.4% of the training cohort and 25.8% of the validation cohort. Sex, age, BMI, hypertension, preoperative eGFR, and remnant kidney proportion (RKP) measured by computerized tomography (CT) volumetry were found significant in the univariable analysis. These variables informed a scoring system based on multivariable analysis: sex (male: 1, female: 0), age at operation (< 30: 0, 30–39: 1, 40–59: 2, ≥ 60: 3), preoperative eGFR (≥ 100: 0, 90–99: 2, 80–89: 4, < 80: 5), and RKP (≥ 52%: 0, < 52%: 1). The total score ranged from 0 to 10. The model showed good discrimination for the primary endpoint in both cohorts. The prediction model provides a useful tool for estimating post-donation renal dysfunction risk, factoring in the side of the donated kidney. It offers potential enhancement to pre-donation evaluations.

## Introduction

Every year, over 35,000 people worldwide undergo donor nephrectomy to provide a kidney for transplantation to a recipient in need^1^. While living donor kidney transplantation (KT) constitutes one-third of all kidney transplants in the United States, the proportion of living donor KTs exceeds 60% in Asian countries, including South Korea^2,3^. Since living kidney donors are undergoing nephrectomy for reasons unrelated to their own health, it is crucial to ensure their long-term safety after the procedure^4,5^. Although kidney donors have a slightly increased risk of end-stage renal disease (ESRD) compared to matched healthy non-donors, the absolute risk remains very low^6,7^. Yet, due to the loss of half the nephrons after nephrectomy, kidney donation may lead to long-term deterioration of kidney function^8,9^.

To minimize risks for kidney donors, appropriate selection of the kidney for transplant through assessment of pre-donation glomerular filtration rate (GFR), differential renal function, and vascular anatomy is essential^10–14^. If renal function is suitable and there are no abnormalities in the kidney parenchyma and vascular structure, the kidney with lower function is typically selected for donation^13,14^. While nuclear renography has traditionally been used to measure differential renal function^14,15^, several studies have proposed methods for estimating split renal function based on kidney volume proportions determined by computerized tomography (CT) volumetry^12,16–18^. Moreover, a recent study suggested that CT volumetry is superior to nuclear renography in predicting postoperative renal function in living kidney donors^19^.

Although predicting the risk of ESRD for individual donor candidates is challenging, multiple demographic and laboratory data can be combined to estimate the long-term risk of ESRD after donation (www.transplantmodels.com/esrdrisk)^20^. However, there are limited studies that focused on predicting individual ESRD risk after donation and estimating post-donation GFR according to the side of donation. Several factors, including race, sex, albuminuria, hypertension, smoking history, and diabetes, are known to contribute to renal dysfunction after kidney donation, but there are few studies comparing the relative risk based on the side of donation^13,14^. It has been reported that CT volumetry is superior to nuclear renography in predicting residual kidney function in living kidney donors^19^. In another study, the clinical characteristics of living donors and remnant kidney volume based on CT volumetry were analyzed to estimate the degree of compensation of the contralateral kidney after donation^21^; however, the number of study patients was small, and the prediction model was not validated with another cohort.

In this study, we aimed to develop and validate a prediction model for post-donation renal function using clinical variables from live kidney donors and the kidney volume measured by CT volumetry.

## Results

### Baseline characteristics

The baseline characteristics of kidney donors in the training and validation cohorts are presented in Table 1. Clinical variables, including the side of donation and remnant kidney volume, were compared between the two cohorts. In the training cohort, female donors (55.7% vs. 50.1%, p = 0.016) and donors with a recent smoking history (24.4% vs. 17.7%, p < 0.001) were more common compared to the validation cohort. Additionally, the training cohort had higher mean values of variables such as systolic blood pressure (124.0 ± 15.4 vs. 116.3 ± 13.5 mmHg, p < 0.001), diastolic blood pressure (80.0 ± 10.1 vs. 69.0 ± 10.0 mmHg, p < 0.001), eGFR (103.6 ± 13.0 vs. 100.5 ± 12.7 mL/min/m^2^, p < 0.001), remnant kidney volume (173.0 ± 30.3 vs. 167.4 ± 32.3 mL, p < 0.001), donated kidney volume (168.5 ± 29.4 vs. 165.6 ± 31.3 mL, p = 0.033), and remnant kidney proportion (50.7 ± 2.2% vs. 50.3 ± 2.9%, p < 0.001). The left kidney was used less commonly in the training cohort compared to the validation cohort (55.4% vs. 65.5%, p < 0.001).

**Table 1:** Baseline characteristics of the training cohort and the validation cohort.

Variables	Training cohort	Validation cohort	*P* Value
Female sex, n (%)	907 (55.7)	346 (50.1)	0.016
Age at operation, year (SD)	44.3 (11.7)	44.4 (12.3)	0.94
Left kidney donation, n (%)	902 (55.4)	452 (65.5)	< 0.001
Height, cm (SD)	164.0 (8.9)	164.7 (8.6)	0.08
Weight, kg (SD)	65.9 (12.1)	66.2 (11.9)	0.58
BMI, kg/m^2^ (SD)	24.4 (3.3)	24.3 (3.2)	0.50
SBP, mmHg (SD)	124 (15.4)	116.3 (13.5)	< 0.001
DBP, mmHg (SD)	80 (10.1)	69 (10.0)	< 0.001
HBV carrier, n (%)	4 (0.2)	7 (1.0)	0.033
Hb A1c, % (SD)	5.50 (0.35)	5.46(0.4)	0.07
eGFR(CKD-Epi), mL/min/m^2^ (SD)	103.6 (13.0)	100.5 (12.7)	< 0.001
< 80, n (%)	66 (4.1)	39 (5.7)	
80 ≤ eGFR < 90, n (%)	160 (9.8)	84 (12.2)	
90 ≤ eGFR < 100, n (%)	349 (21.4)	202 (29.3)	
≥ 100, n (%)	1053 (64.7)	365 (52.9)	
Hypertension medication, n (%)	177 (10.9)	56 (8.1)	0.052
Smoking, n (%)	398 (24.4)	122 (17.7)	< 0.001
Remnant kidney volume, ml (SD)	173.0 (30.3)	167.4 (32.3)	< 0.001
Donation kidney volume, ml (SD)	168.5 (29.4)	165.6 (31.3)	0.033
RKP, % (SD)	50.7 (2.24)	50.3 (2.85)	< 0.001

*BMI* body mass index, *SBP* systolic blood pressure, *DBP* diastolic blood pressure, *eGFR* estimated glomerular filtration rate, *RKP* remnant kidney proportion.

### Univariable and multivariable analysis in the training cohort and prediction scoring model

Univariable logistic regression analysis revealed that the primary endpoint (decrease in eGFR to less than 60 mL/min/1.73 m^2^ at six months post-donation) was significantly associated with male sex (Odds ratio [OR] 2.19, 95% Confidence interval [CI] 1.66–2.92, p < 0.001), age at operation (OR 1.11, 95% CI 1.09–1.13, p < 0.001), BMI (OR 1.07, 95% CI 1.03–1.12, p = 0.001), systolic blood pressure (OR 1.02, 95% CI 1.01–1.03, p < 0.001), diastolic blood pressure (OR 1.02, 95% CI 1.01–1.03, p = 0.008), HbA1c (OR 2.79, 95% CI 1.81–4.32, p < 0.001), eGFR (OR 0.86, 95% CI 0.84–0.88, p < 0.001), hypertension (OR 2.60, 95% CI 1.80–3.72, p < 0.001), remnant kidney volume (OR 0.99, 95% CI 0.98–0.99, p < 0.001), donated kidney volume (OR 0.99, 95% CI 0.99–1.00, p < 0.001), and remnant kidney proportion (OR 0.87, 95% CI 0.82–0.93, p < 0.001) (Table 2). Multivariable logistic regression analysis showed that male sex, older age at operation, lower eGFR before donation, and smaller RKP were independently associated with the primary endpoint (Table 2).

**Table 2:** Univariable and multivariable logistic regression in the training cohort.

Variables	Patients (n)	Events (n)	Univariable		Multivariable		
			OR (95% CI)	*P* value	OR (95% CI)	β	*P* value
Male sex (vs. female sex)	721	143	2.19 (1.66–2.92)	< 0.001	2.27 (1.59–3.27)	0.822	< 0.001
Age at operation (per 1-year increase)			1.11 (1.09–1.13)	< 0.001	1.06 (1.04–1.08)	0.061	< 0.001
Left-side donation (vs. right)	902	138	1.17 (0.89–1.55)	0.27			
Height			1.01 (1.00–1.03)	0.16			
Weight			1.02 (1.01–1.03)	0.001			
BMI (per 1-kg/m^2^ increase)			1.07 (1.03–1.12)	0.001			
SBP (per 1-mmHg increase)			1.02 (1.01–1.03)	< 0.001			
DBP (per 1-mmHg increase)			1.02 (1.01–1.03)	0.008			
Hb A1c (1% increase)			2.79 (1.81–4.32)	< 0.001			
eGFR (1 mL/min/1.72 m^2^ increase)			0.86 (0.84–0.88)	< 0.001			
eGFR (≥ 100)	1053	26	Reference	< 0.001	Reference		< 0.001
eGFR (90–100 versus ≥ 100)	349	77	11.18 (7.12–18.09)	< 0.001	6.29 (3.86–10.52)	1.840	< 0.001
eGFR (80–90 versus ≥ 100)	160	80	39.5 (24.34–66.03)	< 0.001	23.85 (14.24–41.03)	3.172	< 0.001
eGFR (< 80 versus ≥ 100)	66	52	146.71 (74.38–307.75)	< 0.001	70.29 (34.09–153.06)	4.253	< 0.001
Hypertension	177	49	2.60 (1.80–3.72)	< 0.001	–		–
Smoking	398	58	1.01 (0.73–1.39)	0.93	–		–
Remnant kidney volume (per 1-cm^3^ increase)			0.99 (0.98–0.99)	< 0.001	–		–
Donation kidney volume (per 1-cm^3^ increase)			0.99 (0.99–1.00)	< 0.001	–		–
RKP (per 1% increase)			0.87 (0.82–0.93)	< 0.001	0.83 (0.76–0.90)	**− **0.186	< 0.001
Volume difference (per 1-cm^3^ increase)			1.02 (1.01–1.03)	< 0.001	–		–

*BMI* body mass index, *SBP* systolic blood pressure, *DBP* diastolic blood pressure, *eGFR* estimated glomerular filtration rate, *RKP* remnant kidney proportion.

**Figure 1:**
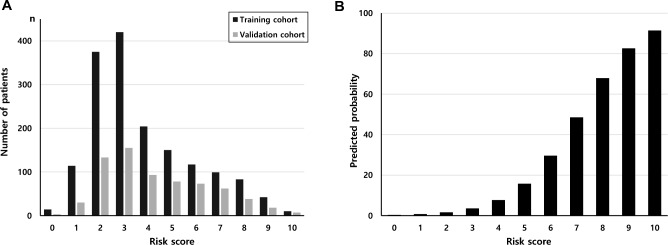
The total number of donors according to the risk score (**A**) and the probability of occurrence of the primary endpoint for each score (**B**) in the training cohort.

Based on the results of multivariable analysis, we used four variables—sex, age at operation, eGFR, and RKP to stratify the risk of renal dysfunction after kidney donation; specifically, we divided the three continuous variables (age, eGFR, RKP) into five, four, and three categories, respectively (Table 3). We assigned a point of 1 for male sex, and the points for other variables were assigned using the rounded quotient of *β *(*W − W*_*REF*_)/*β*_*male*_. The risk score is obtained by summing the points for four variables. The total number of donors according to the risk score in the training cohort is shown as blue bars on the histogram in Fig. 1A.

**Table 3:** Risk scores for event occurrence in living kidney donors.

Risk factor	Categories	Reference value (*W*)	*β*_*penalized*_	*β *(*W − W*_*REF*_)	Points = *β *(*W − W*_*REF*_)/*β*_*male*_
Sex					
	F	0		0.000	0
	M	1	0.8083	0.8083	1
Age at operation, years			0.0601		
	< 30	24.0		0.000	0
	30–39	34.5		0.631	1
	40–49	44.5		1.232	2
	50–59	55.5		1.893	2
	≥ 60	63.2		2.356	3
eGFR(CKD-EPI), mL/min/m^2^					
	≥ 100			0.000	0
	90–100		1.7759	1.776	2
	80–90		3.1002	3.100	4
	< 80		4.1658	4.166	5
RKP, %			**− **0.181		
	≥ 52	47.9		0.000	0
	49–52	50.5		0.543	1
	< 49	53.5		1.023	1

*eGFR* estimated glomerular filtration rate, *RKP* remnant kidney proportion.

**Figure 2:**
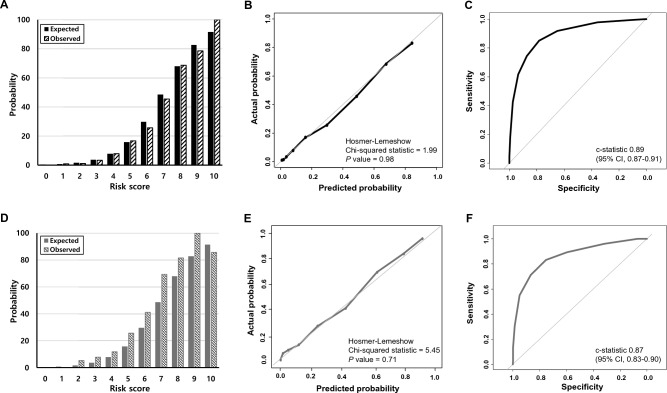
The performance of the score-based prediction system in the training cohort (**A**–**C**) and the validation cohort (**D**–**F**). Observed and expected probabilities of the primary endpoint in the training cohort (**A**,**D**). The calibration according to the Hosmer–Lemeshow test (**B**,**E**). Receiver operating characteristic curves of the score-based prediction system to predict the primary endpoint (**C**,**F**).

The probability of occurrence of the primary endpoint was calculated for each score (Fig. 1B). The probability of occurrence of the primary endpoint increased steeply as the risk score increased. Specifically, while the predicted probability of the primary endpoint was less than 8% when the total score was less than 5, the probability increased to over 48% when the total score was more than 6 (Fig. 1B). The performance of this score-based prediction system using four variables was excellent in the training cohort (Fig. 2). The expected probabilities were similar to the observed probabilities (Fig. 2A), and the calibration according to the Hosmer–Lemeshow test showed considerable concordance (chi-squared statistic = 1.99, *P* value = 0.98) (Fig. 2B). The prediction model had good discriminative capacity, with a c-statistic value of 0.89 (95% CI 0.87–0.91) (Fig. 2C).

### Validation cohort

To assess the validity of the prediction model, the scoring system was applied to an external validation cohort. Although the observed probability was higher than the expected probability at the risk score of 9, there was a strong overall similarity between the observed and expected probabilities in the validation cohort (Fig. 2D). While the calibration was lower compared to the training cohort, the Hosmer–Lemeshow test still demonstrated good concordance (chi-squared statistic = 5.45, *P* value = 0.71) (Fig. 2E). Furthermore, the score-based prediction model performed well in terms of discrimination in the validation cohort as well (c-statistic 0.87, 95% CI 0.83–0.90) (Fig. 2F).

## Discussion

In this study, we developed a simple prediction model comprising four variables (sex, age, eGFR, RKP) for renal deterioration after donor nephrectomy and validated the scoring system externally using an independent external cohort. Postoperative eGFR can be predicted by donor age, sex, preoperative eGFR, and RKP. Scores were assigned based on the degree to which each factor was related to the occurrence of the primary endpoint. Among the factors, preoperative eGFR and age had more significant influences on the primary endpoint. Unlike previous studies, RKP, determined by kidney volume from CT volumetry, was included as an adjustable factor to predict renal deterioration after donor nephrectomy. In other words, preoperative measurement of RKP by CT volumetry may be able to assist surgeons in deciding which kidney is more suitable for donation in order to preserve renal function as much as possible after donor nephrectomy. We expect that this scoring system will be useful for estimating renal function after donor nephrectomy.

Several studies have estimated the risk of ESRD in kidney donors. Grams et al. suggested that the long-term risk of ESRD in kidney donor candidates can be estimated using multiple demographic and health characteristics^20^; however, their model is designed to predict the ESRD risk if a person does not donate a kidney. In other words, the model does not predict kidney function after donation, and a total of 10 factors were used for prediction, which cannot be adjusted unless the donor is changed. Okumura et al. suggested a compensation prediction score using four factors, including age, sex, history of hypertension, and the ratio of the remnant kidney volume to body weight^21^. They defined favorable compensation as post-donation eGFR at 1 year being more than 60% of the pre-donation eGFR. However, for this study, only 133 living donors were enrolled at a single center and there was no external validation. Rook et al. predicted post-donation renal function impairment, defined as a GFR of ≤ 60 mL/min/1.73 m^2^, using pre-donation eGFR, BMI, and age^22^; however, this study evaluated only 125 donors from a single center and there was no external validation as well. Furthermore, they did not measure remnant kidney volume from CT volumetry.

It has already been reported that age, male sex, and lower preoperative eGFR are significantly associated with renal dysfunction and ESRD after donor nephrectomy^21,22^. However, these factors cannot be adjusted for the purpose of reducing the risk of ESRD. Therefore, we believe it is important that RKP was found as a significant factor in multivariable logistic regression analysis because RKP is the only adjustable factor for a live kidney donor candidate. The predicted probability of the primary endpoint increased steeply with a 1-point increase in the scoring system, especially when the total risk score was more than 5 (Fig. 1B). In other words, in donors with a higher total risk score, the RKP should be strictly preserved by selecting the smaller kidney for donation.

There are some limitations to this study. This was a retrospective observational study with possible selection biases and confounders. Nevertheless, the prediction model for post-donation renal function developed based on the cohort of 1628 patients showed robust results in external validation with 690 patients. Another limitation is that the scoring system was based on eGFR at 6 months post-donation rather than long-term clinical outcomes. However, it has been known that eGFR at 6 months post-donation is useful for predicting long-term renal function after kidney donation^23^. In addition, there might be a certain amount of error in kidney volume measurement between the training and validation cohorts. It should also be considered that we measured the total volume of each kidney rather than the volume of the cortex, which more directly reflects renal function.

In conclusion, we developed a simple scoring system comprising four variables (sex, age, eGFR, RKP) to predict renal function after living donor nephrectomy and validated its robustness in an independent external cohort. We expect that this model would be useful for estimating the risk of post-donation renal dysfunction and for determining the more appropriate side of the kidney to ensure the safety of kidney donors.

## Methods

### Study population and data sources

We conducted a multicenter retrospective cohort study involving adult patients (≥ 18 years old) who underwent donor nephrectomy between May 2005 and December 2019 at two tertiary referral centers (Asan Medical Center and Samsung Medical Center) in South Korea. Approval from the institutional review board (IRB) was obtained at each center (Approval numbers: Asan Medical Center IRB 2021-0465, Samsung Medical Center IRB 2021-07-013-001). Asan Medical Center IRB and Samsung Medical Center IRB waived written informed consent because of the retrospective and noninvasive nature of this study. clinical and research activities being reported are consistent with the Principles of the Declaration of Istanbul as outlined in the 'Declaration of Istanbul on Organ Trafficking and Transplant Tourism'. A total of 4295 individuals underwent donor nephrectomy at the two centers during the study period, among whom we excluded the following donors: (1) donors who did not have dynamic CT kidney volumetry before donor nephrectomy (n = 994), (2) donors who did not have postoperative serum creatinine measured 4–8 months following donor nephrectomy (n = 781), (3) donors with one or more renal stones (n = 198), and (4) donors with complicated cysts (more than IIF category by the Bosniak classification^24^) (n = 4). Finally, the study included 1628 patients in the training cohort (Asan Medical Center) and 690 patients in the validation cohort (Samsung Medical Center).

The primary endpoint was a decrease in estimated GFR (eGFR) to less than 60 mL/min/1.73 m^2^ at 6 months post-donation. This endpoint was determined based on previous reports in which chronic kidney disease was defined as kidney damage or glomerular filtration rate < 60 mL/min/1.73 m^2^ for three months or more^25^. And early post-donation renal function was associated with the subsequent risk of ESRD in living kidney donors^23^. Specifically, eGFR measured 6 months after donation was independently associated with ESRD risk, even after adjusting for pre-donation characteristics.

We investigated several factors that are known to be associated with decreased eGFR in living kidney donors, including sex, age, body mass index (BMI), blood pressure, hemoglobin A1c, preoperative eGFR, hypertension medication, and smoking history^20^. Remnant kidney proportion (RKP) was defined as the proportional remnant kidney volume per total kidney volume, measured by dynamic CT kidney volumetry (RKP = remnant kidney volume (mL)/total kidney volume (mL)).

### Decision of donation side and postoperative management

The decision regarding which kidney to donate was made after considering factors such as vasculature, preoperative eGFR, split renal function assessed by renal scintigraphy (technetium-99m diethylenetriaminepentaacetic acid or Tc-99m dimercaptosuccinic acid), kidney volume measured by CT volumetry, and the presence of atypical or large renal cysts. If there was a considerable difference in the relative function determined by renal scintigraphy or kidney volume measured by CT volumetry between the two kidneys, the one with inferior function was selected for donation. A kidney with calcification or stenosis of the renal artery was also considered for donation. The left kidney was selected if there was no significant difference between the two kidneys.

Donors underwent hand-assisted laparoscopic surgery for nephrectomy and were discharged five days after surgery. Renal function was assessed using eGFR, obtained through the Chronic Kidney Disease Epidemiology Collaboration equation, at 1 week, 1 month, 3 months, 6 months, and 1 year after surgery.

### Kidney volume measurement

Preoperative CT scans were performed using a 16- or 64-multidetector CT scanner (LightSpeed 16 or Optima CT660, GE Healthcare; Somatom Sensation 16, Siemens Healthcare). The scanning protocol consisted of three phases: unenhanced phase, corticomedullary phase (30 s after contrast injection), and nephrographic phase (90 s after contrast injection). The scanning parameters were as follows: pitch, 1.5; tube voltage, 120 kV; tube current, 210–240 mA; and slice thickness, 3–5 mm.

Kidney volume for each patient was measured using the GE Advantage Windows Workstation (version 3.0; General Electric Medical Systems, Milwaukee, WI, USA) in the training cohort, and the Aquaris iNtuition Viewer version 4.4.13 (TeraRecon; Durham, NC, USA) in the validation cohort. Kidney length was measured using coronal sections, and kidney volume was determined from contiguous slices. In coronal section images with parenchymal enhancement, the region of interest was drawn around the kidney, and slices were reconstructed at 1-mm intervals to obtain a 3D volume-rendered image of the kidney. Volume was calculated by multiplying the sum of areas from each slice by the reconstruction interval at the workstation.

### Statistical analysis

Statistical analysis was performed using R Statistics ver. 4.04. Continuous variables were compared using the t-test, while categorical variables were compared using the Chi-squared test or Fisher's exact test, as appropriate. Clinical variables for event prediction were entered into the univariate analysis. To identify factors independently related to an event, a bootstrap statistical technique was used. Resampling was performed 1000 times, and backward elimination was conducted. Only factors present in more than 50% of the 1000 samples were selected as final items. A multivariable analysis was performed with the final variables, and the coefficient of each variable was incorporated into a scoring system. This point-scoring algorithm was applied to patients in the validation cohort, and the risk score was derived by summing the points corresponding to each variable. The scoring system was out of 10, and the probability of event occurrence for each score was calculated. To evaluate the performance of the scoring system, we used the Hosmer and Lemeshow goodness-of-fit (GOF) test, receiver operating characteristic (ROC) curves, and calibration curves.

## Data Availability

The datasets generated and/or analyzed during the current study are available from the corresponding author on reasonable request.
